# Optimized selection of specific antibodies against rodent ESR1 proteins and their application for immunohistochemistry and dual immunohistofluorescence with the specific anti-ESR2 antibody PPZ0506

**DOI:** 10.1007/s00418-025-02454-6

**Published:** 2026-01-16

**Authors:** Mika Soma, Masahiro Morishita, Shimpei Higo, Kenta Sekiya, Hirotaka Ishii

**Affiliations:** 1 Department of Anatomy and Neurobiology, Graduate School of Medicine, Nippon Medical School, 1-1-5 Sendagi, Bunkyo-Ku, Tokyo, 113-8602 Japan; 2 Present Address: Department of Anatomy and Neuroscience, Graduate School of Medical Sciences, Kyushu University, 3-1-1 Maidashi, Higashi-Ku, Fukuoka, 812-8582 Japan

**Keywords:** Antibody validation, Dual immunohistofluorescence, ESR1, ESR2, Immunohistochemistry, PPZ0506

## Abstract

The development of immunohistochemical and immunohistofluorescence assays is essential for investigating the tissue and cellular distribution of target proteins. In this study, we identify specific anti-ESR1 antibodies against rodent ESR1 proteins and evaluate their applicability for immunohistochemistry and dual immunohistofluorescence with the specific anti-ESR2 antibody PPZ0506. We assessed the specificity and cross-reactivity of six commercially available anti-ESR1 antibodies (Clones MC-20, C1355, E115, H4624, SP1, and F-10) against mouse and rat ESR1 proteins using immunoblotting and immunocytofluorescence assays. Among them, MC-20, C1355, E115, and H4624 exhibited specific immunoreactivity to mouse and rat ESR1 proteins. These four antibodies were subsequently applied to paraffin-embedded ovarian and uterine sections from mice and rats. Heat-induced antigen retrieval and an appropriate antibody dilution were required to obtain specific and adequate signals. MC-20 and E115 were suitable for immunohistochemical detection of ESR1 proteins, while C1355 was effective for uterine tissue staining. H4624 showed utility only in mouse tissues. Furthermore, rabbit-derived MC-20 and E115 antibodies were successfully employed in dual immunohistofluorescence assays with the mouse monoclonal PPZ0506 antibody, enabling simultaneous visualization of ESR1 and ESR2 proteins in paraffin-embedded ovarian sections. Notably, little cellular co-localization of ESR1 and ESR2 proteins was observed in mouse and rat ovarian sections. These findings provide a validated set of antibodies for ESR1 immunohistochemical detection and demonstrate their compatibility with ESR2 co-labeling, facilitating detailed analysis of estrogen receptor distribution in rodent tissues.

## Introduction

The ovarian steroid hormones estrogens regulate a broad spectrum of physiological processes in both reproductive and non-reproductive organs (Nilsson et al. 2001). Beyond their essential roles in normal development and homeostasis, estrogens are implicated in various pathophysiological conditions, including modulation of cell proliferation and progression of estrogen-sensitive cancers. These effects are mediated by estrogen receptors (ERs), which function as ligand-activated transcription factors within the nuclear receptor superfamily (Hewitt and Korach 2018). Two distinct ER subtypes have been identified in mammals: estrogen receptor α (ESR1; also known as ERα) and estrogen receptor β (ESR2; ERβ).

Immunoassays, particularly those employing immunohistochemistry or immunohistofluorescence, are indispensable tools in clinical diagnostics and basic biomedical research (Kohale et al. 2023). ESR1 is a widely used diagnostic marker in breast cancer pathology (Masuda and Nakanishi 2023), and several anti-human ESR1 antibodies have been developed for this purpose. In rodent studies, antibodies targeting rodent ESR1 proteins have been employed to investigate their tissue and cellular localization. Among these, the polyclonal antibody MC-20, which recognizes both mouse and rat ESR1 proteins, has been extensively used for ESR1 immunohistochemistry and immunohistofluorescence (Islam et al. 2012; Kunimura et al. 2024; Matsuda and Tanaka 2025; Mori et al. 2008; Saji et al. 2000). However, the discontinuation of MC-20 has created an urgent need to identify alternative antibodies with high specificity and sensitivity for rodent ESR1 proteins, as well as to optimize corresponding immunostaining protocols.

In contrast to ESR1, research on ESR2 has long been hindered by the absence of reliable anti-ESR2 antibodies. This limitation was recently addressed with the discovery of PPZ0506, a mouse monoclonal antibody that specifically recognizes both human and rodent ESR2 proteins (Andersson et al. 2017; Ishii et al. 2019). Successful application of PPZ0506 in immunohistochemistry enables more precise analysis of ESR2 expression and localization (Andersson et al 2017; Hattori et al. 2021; Hawse et al. 2020; Ishii et al. 2019; Morishita et al. 2023a, b; Ozawa et al. 2022; Schröder et al. 2022). Since ESR1 and ESR2 form heterodimers that modulate each other’s activity in vitro (Cowley et al. 1997; Pettersson et al. 1997), studying their co-expression patterns in situ is essential to understanding the intricate mechanisms of estrogen signaling. Therefore, establishing a robust dual immunohistofluorescence protocol using specific antibodies against both ESR1 and ESR2 proteins is of considerable interest in estrogen research.

In this study, we systematically evaluated the specificity of several commercially available anti-ESR1 antibodies for detecting mouse and rat ESR1 proteins using immunoblotting and immunocytofluorescence. On the basis of these results, we optimized immunohistochemistry protocols for detecting ESR1 proteins in rodents. Additionally, we developed a dual immunohistofluorescence method to simultaneously visualize ESR1 and ESR2 proteins in rodent ovaries using the selected anti-ESR1 antibodies in conjunction with the anti-ESR2 antibody PPZ0506.

## Materials and methods

### Chemicals

All chemicals used in this study were purchased from Fujifilm (Tokyo, Japan), unless otherwise stated.

### Antibodies

The anti-mouse ESR1 rabbit polyclonal antibody (Clone MC-20; Cat. No. sc-542; RRID: AB_631470) and the anti-human ESR1 rabbit polyclonal antibody (Clone C1355; Cat. No. 06-935; RRID: AB_310305) were obtained from Santa Cruz Biotechnology (Dallas, TX, USA) and Merck (Darmstadt, Germany), respectively. The anti-human ESR1 mouse monoclonal antibody (Clone H4624; Cat. No. PP-H4624-00; RRID: AB_604960) and the anti-human ESR1 rabbit monoclonal antibodies (Clones E115; Cat. No. ab32063; RRID: AB_732249, and SP1; Cat. No. ab16660; RRID: AB_443420) were purchased from Perseus Proteomics (Tokyo, Japan) and Abcam (Cambridge, UK), respectively. The anti-human ESR1 mouse monoclonal antibody (Clone F-10; Cat. No. sc-8002; RRID: AB_627558) was also obtained from Santa Cruz Biotechnology. The anti-human ESR2 mouse monoclonal antibody (Clone PPZ0506; Cat. No. PP-PPZ0506-00; RRID: AB_604962) and the anti-DYKDDDDK mouse monoclonal antibody (Clone 2H8; Cat. No. KAL-KO602-S; RRID: AB_3714826) were purchased from Perseus Proteomics and Medical Chemistry Pharmaceutical (Hokkaido, Japan), respectively. Detailed information on all primary antibodies used in this study is provided in Table 1.

**Table 1:** Primary antibody information

Antibody	Manufacturer	Immunogen	Host	Catalog No.
			Clonality	RRID
ESR1				
MC-20	Santa Cruz Biotechnology	Mouse ESR1	Rabbit	sc-542
		580–599 aa	Polyclonal	AB_631470
C1355	Merck	Rat ESR1	Rabbit	06–935
		586–600 aa	Polyclonal	AB_310305
E115	Abcam	Human ESR1	Rabbit	ab32063
		50–150 aa	Monoclonal	AB_732249
H4624	Perseus Proteomics	Human ESR1	Mouse	PP-H4624-00
		2–180 aa	Monoclonal	AB_604960
SP1	Abcam	Human ESR1	Rabbit	ab16660
		C-terminus	Monoclonal	AB_443420
F-10	Santa Cruz Biotechnology	Human ESR1	Mouse	sc-8002
		576–595 aa	Monoclonal	AB_627558
ESR2				
PPZ0506	Perseus Proteomics	Human ESR2	Mouse	PP-PPZ0506-00
		2–88 aa	Monoclonal	AB_604962
FLAG tag				
2H8	Medical Chemistry Pharmaceutical	DYKDDDDK tag	Mouse	KAL-KO602-S
		(N-terminal)	Monoclonal	AB_3714826

*aa* amino acid

Horseradish peroxidase (HRP)-conjugated secondary antibodies (Anti-rabbit IgG, HRP-linked Antibody; Cat. No. 7074; RRID: AB_2099233, and Anti-mouse IgG, HRP-linked Antibody; Cat. No. 7076; RRID: AB_330924) were obtained from Cell Signaling Technology (Danvers, MA, USA). Alexa Fluor 488-conjugated donkey anti-IgG secondary antibodies [Donkey Anti-Rabbit IgG (H + L), Highly Cross-Adsorbed Secondary Antibody, Alexa Fluor 488; Cat. No. A-21206; RRID: AB_2535792, and Donkey Anti-Mouse IgG (H + L), Highly Cross-Adsorbed Secondary Antibody, Alexa Fluor 488; Cat. No. A-21202; RRID: AB_141607], as well as the Alexa Fluor 568-conjugated donkey anti-mouse IgG secondary antibody [Donkey Anti-Mouse IgG (H + L), Highly Cross-Adsorbed Secondary Antibody, Alexa Fluor 568; Cat. No. A10037; RRID: AB_11180865], were purchased from Thermo Fisher Scientific (Waltham, MA, USA). HRP-polymer secondary antibodies [Goat Anti-Mouse IgG H&L (HRP polymer); Cat. No. ab214879; RRID: AB_3678671, and Goat Anti-Rabbit IgG H&L (HRP polymer); Cat. No. ab214880; RRID: AB_3106917] were obtained from Abcam.

### Cell cultures

The COS-7 cell line (JCRB No. JCRB9127), derived from African green monkey kidney fibroblast-like cells, was obtained from the Japanese Collection of Research Bioresources Cell Bank (Osaka, Japan). The cell line was authenticated and confirmed to be free of mycoplasma contamination. Because COS-7 cells exhibit strong adhesion properties and lack endogenous ER activity, they were used as host cells for the transient, induced expression of ERs via transfection, as described in our previous studies (Ishii et al. 2017, 2019). COS-7 cells were maintained in phenol red-free, high-glucose Dulbecco’s Modified Eagle Medium (Fujifilm) supplemented with 10% fetal bovine serum (Cytiva, Marlborough, MA, USA) and 1 × penicillin–streptomycin solution (Fujifilm). Cultures were incubated at 37 °C in a humidified atmosphere containing 5% CO_2_ and 95% air. Cells were routinely passaged every 3–4 days and used for experiments within 15 passages.

### Plasmid vectors

Expression vectors encoding full-length ESR1 proteins (pcDNA-human ESR1, pcDNA-mouse ESR1, pcDNA-rat ESR1, and pFLAG-human ESR1) were constructed in our previous studies (Hattori et al. 2016; Ishii et al. 2017, 2019). The coding sequences of mouse and rat *Esr1* were amplified from the pcDNA-mouse ESR1 and pcDNA-rat ESR1 vectors, respectively, using KOD-Plus-NEO polymerase (Toyobo, Osaka, Japan) with primers containing restriction site adaptors. Amplified products were cloned into pCMV-Tag 2B vectors (Agilent Technologies, Santa Clara, CA, USA) and fused in-frame with FLAG epitope tag sequences, resulting in pFLAG-mouse ESR1 and pFLAG-rat ESR1 constructs. All cloned fragments were verified by DNA sequencing.

### Transfection and immunoblotting

COS-7 cells were cultured in 60-mm dishes (AGC Techno Glass, Shizuoka, Japan) and transfected with 1 μg of plasmid DNA per dish (pFLAG-human ESR1, pFLAG-mouse ESR1, or pFLAG-rat ESR1) using FuGENE HD Transfection Reagent (Promega, Madison, WI, USA) according to the manufacturer’s instructions. Cells transfected with an empty vector or mock-transfected cells were used as negative controls. At 48 h post-transfection, cells were washed with phosphate-buffered saline (PBS) and lysed in RIPA Buffer (Fujifilm). Lysates were briefly sonicated before determining protein concentrations. Equal amounts of proteins were mixed with 4 × sample buffer [Sample Buffer Solution with 3-Mercapto-1,2-propanediol (× 4); Fujifilm] and heated at 96 °C for 5 min.

Samples were loaded onto 10–20% gradient polyacrylamide gels (e-PAGEL E-T1020L; ATTO Corporation, Tokyo, Japan) and separated by electrophoresis, followed by electrophoretic transfer onto polyvinylidene difluoride membranes (ClearBlot P plus membrane; ATTO Corporation). Precision Plus Protein Dual Color Standards (Bio-Rad Laboratories, Hercules, CA, USA) were used to verify electrophoresis and transfer efficiency. Membranes were incubated with the respective anti-ESR1 antibodies at a dilution of 1:1000, followed by HRP-conjugated secondary antibodies at a dilution of 1:2500. Immunoreactive bands were visualized using the ImmunoStar Zeta chemiluminescent substrate (Fujifilm) and imaged with an Amersham ImageQuant 800 system (Cytiva). Luminescent signals were overlaid on membrane images, and image contrast and brightness were adjusted using ImageQuant TL software (version 10.0; Cytiva).

### Transfection and immunocytofluorescence

COS-7 cells were cultured on collagen-coated glass-bottom dishes (Matsnami Glass, Osaka, Japan) and transfected with 500 ng of plasmid DNA per dish (pcDNA-human ESR1, pcDNA-mouse ESR1, or pcDNA-rat ESR1) using FuGENE HD Transfection Reagent according to the manufacturer’s instructions. Mock-transfected cells were used as negative controls. At 48 h post-transfection, cells were washed with PBS and fixed with 10% formalin in PBS for 30 min at room temperature.

Immunocytofluorescence was performed as previously described (Ishii et al. 2019). Anti-ESR1 primary antibodies and Alexa Fluor 488-conjugated donkey anti-IgG secondary antibodies were used at a dilution of 1:250. Nuclei were counterstained with 4′,6-diamidino-2-phenylindole (DAPI; Fujifilm).

Fluorescence signals were acquired using a BZ-8000 All-in-One fluorescence microscope (Keyence Corporation, Osaka, Japan) equipped with a Nikon Plan Apo objective lens (×20/0.75; Nikon Corporation, Tokyo, Japan) and Keyence fluorescence filter sets (GFP-B and DAPI; Keyence Corporation). Images were captured at a resolution of 1360 × 1024 pixels and presented in pseudocolor (green for Alexa Fluor 488 and magenta for DAPI).

### Animals and tissue slice preparation

All animal experimental procedures were approved by the Animal Care and Use Committee of Nippon Medical School and conducted in accordance with institutional guidelines (Approval No. 2020-032). Adult female C57BL/6 mice and Wistar rats were obtained from Tokyo Laboratory Animals Science (Tokyo, Japan). Animals were housed in a temperature-controlled room (22–24 °C) under a 12-h light/12-h dark cycle, with standard chow and tap water provided ad libitum. Six mice and four rats aged 10–13 weeks were used. The estrous cycle stage was determined by vaginal smear cytology.

Female mice and rats in the diestrus stage were briefly anesthetized with isoflurane (Isoflurane Inhalation Solution [Pfizer]; Pfizer Japan, Tokyo, Japan) and then deeply anesthetized via intraperitoneal injection of medetomidine hydrochloride (Domitor; Nippon Zenyaku Kogyo, Fukushima, Japan), midazolam (Midazolam Sandoz; Sandoz, Tokyo, Japan), and butorphanol tartrate (Vetorphale; Meiji Seika Pharma, Tokyo, Japan). For tissue fixation, animals were transcardially perfused under deep anesthesia with 0.9% (w/v) saline, followed by 4% (w/v) paraformaldehyde in 0.1 M phosphate buffer (pH 7.4). Organs were then removed and postfixed in the same fixative for 18–24 h at 4 °C.

For paraffin embedding, postfixed tissues were dehydrated through a graded ethanol series, cleared in xylene, and embedded in paraffin (Merck). Sections were cut at 5 μm using a Leica RM2235 rotary microtome (Leica Biosystems, Wetzlar, Germany) and mounted on CREST-coated glass slides (Matsunami Glass). Sections were deparaffinized and rehydrated prior to staining.

### Immunohistochemistry

Mounted tissue sections were treated with 0.3% (v/v) hydrogen peroxide in methanol to quench endogenous peroxidase activity, followed by washing with distilled water. Heat-induced antigen retrieval (HIAR) was performed by incubating sections in 10 mM citrate buffer (pH 6.0) at 121 °C for 10 min using a high-pressure steam sterilizer (LBS-245; Tomy Seiko, Tokyo, Japan).

To block nonspecific binding, Mouse-on-Mouse Blocking Buffer (Abcam) was applied to mouse sections, and 5% normal goat serum (Abcam) diluted in PBS containing 0.3% (v/v) Triton X-100 (PBST) was applied to rat sections. Blocking was performed for 30 min (mouse) or 1 h (rat) at room temperature. Sections were then incubated with anti-ESR1 primary antibodies diluted in PBST for 18 h at 4 °C.

Immunoreactive signals were visualized using HRP-polymer secondary antibodies and 3,3′-diaminobenzidine tetrahydrochloride (Merck) with 0.01% hydrogen peroxide. Sections were counterstained with hematoxylin, dehydrated, and mounted using Permount Mounting Medium (Thermo Fisher Scientific).

### Dual immunohistofluorescence

Mounted ovarian sections were subjected to HIAR in 10 mM citrate buffer (pH 6.0) at 121 °C for 10 min. Following rinsing with PBS, sections were transferred to an acrylate container filled with PBS containing 4.5% H_2_O_2_ and 20 mM NaOH, and bleached for 1 h under LED illumination using a TiYO autofluorescence quenching illuminator (Nepa Gene, Chiba, Japan).

To block non-specific binding, sections were incubated in Mouse-on-Mouse Blocking Buffer for mouse sections or PBST with 5% normal goat serum for rat sections. Subsequently, immunoreactions were performed in PBST at 4 °C for 18 h using rabbit-derived anti-ESR1 antibodies (Clones MC-20 and E115) and mouse monoclonal anti-ESR2 antibody (Clone PPZ0506).

Immunoreactive signals were detected using an Alexa Fluor 488-conjugated donkey anti-rabbit IgG secondary antibody and an Alexa Fluor 568-conjugated donkey anti-mouse IgG secondary antibody. Cell nuclei were counterstained with DAPI. After final PBS washes, sections were mounted with Fluoromount (Diagnostic BioSystems, Pleasanton, CA, USA).

### Microscopic image acquisition

Fluorescent and non-fluorescent images were acquired using an Olympus BX-51 light microscope (Olympus Corporation, Tokyo, Japan) equipped with either a ×10 (UPlanSApo ×10/0.40) or ×20 (UPlanSApo ×20/0.75) objective lens (Olympus Corporation). Images were captured at a resolution of 1600 × 1200 pixels in 8-bit RGB color mode using an Olympus DP-73 digital camera (Olympus Corporation) and Olympus CellSense Standard 1.6 image acquisition software (Olympus Corporation). Immunofluorescent images were obtained using a mercury lamp light source and Olympus fluorescence filter sets (NIBA, WIG, and WU; Olympus Corporation). Fluorescence images from each filter were merged into composite images using Affinity Photo 2 (Serif, Nottingham, UK), with the red channel converted to magenta pseudocolor.

## Results

### Antibody validation

**Fig. 1:**
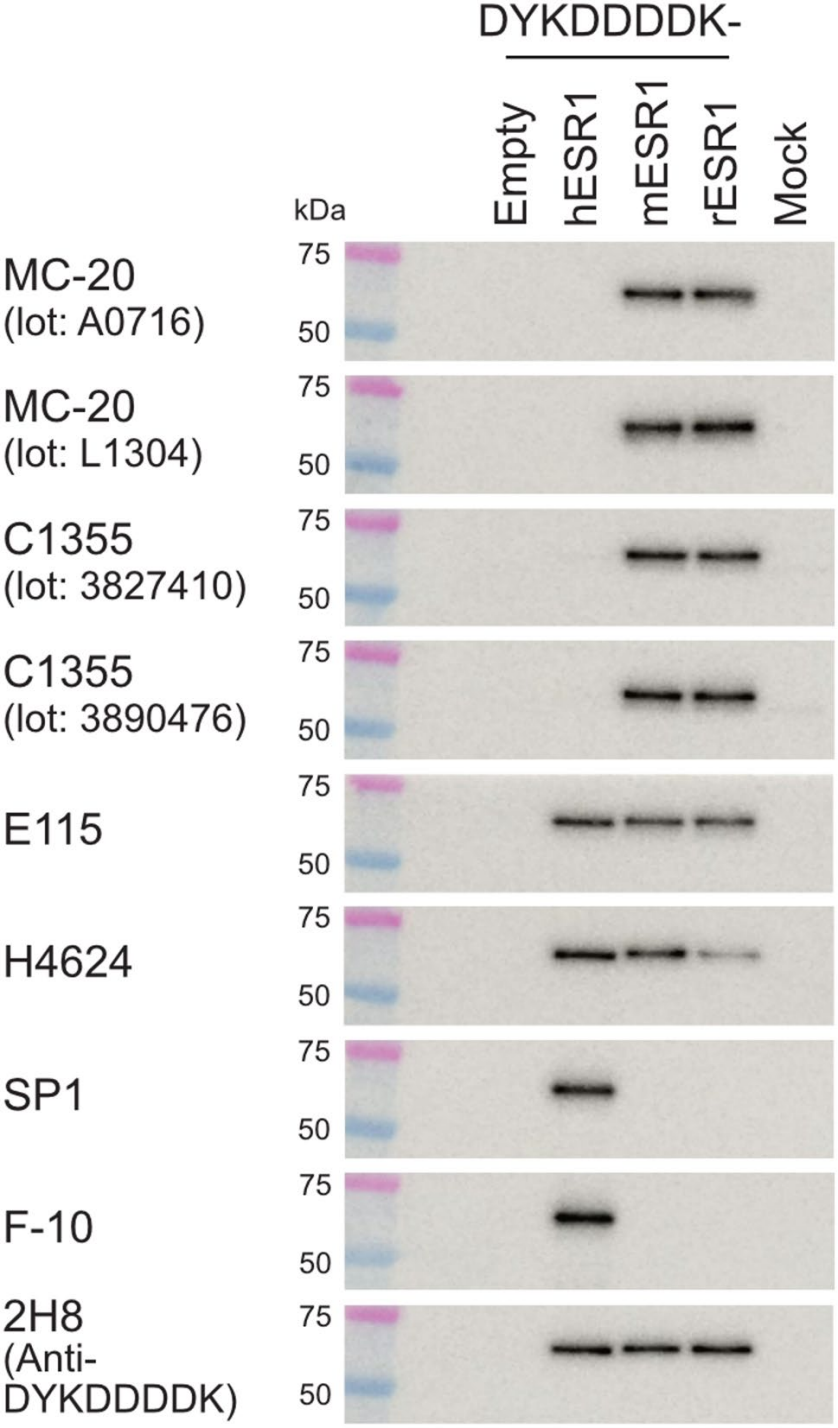
Verification of specific immunoreactivity of anti-ESR1 antibodies against human, mouse, and rat ESR1 proteins by immunoblotting. Immunoblot analysis of FLAG (DYKDDDDK)-tagged ESR1 proteins expressed in transfected COS-7 cells using anti-ESR1 antibodies (Clones MC-20, C1355, E115, H4624, SP1, and F-10) and an anti-DYKDDDDK antibody (Clone 2H8). Two different lots were tested for each polyclonal antibody: MC-20 (Lot Nos. A0716 and L1304) and C1355 (Lot Nos. 3827410 and 3890476). “h,” “m,” and “r” denote human, mouse, and rat, respectively. Cells transfected with empty vectors (empty) and mock-transfected cells (mock) served as negative controls. Equal amounts of protein lysate were loaded per lane (0.5 μg/lane). Precision Plus Protein Dual Color Standards were used as molecular weight markers. Immunoluminescence signals were overlaid on bright-field images of the membranes. Representative images were obtained from the same set of samples. Similar results were observed in four separate experiments (*n* = 4)

Immunoblot analyses were performed to evaluate the specificity and cross-reactivity of commercially available anti-ESR1 antibodies (Clones MC-20, C1355, E115, H4624, SP1, and F-10) against human, mouse, and rat ESR1 proteins (Fig. 1). For each polyclonal antibody, two different lots were tested: MC-20 (Lot Nos. A0716 and L1304) and C1355 (Lot Nos. 3827410 and 3890476). Expression vectors encoding FLAG-tagged ESR1 proteins were transfected into COS-7 cells, and immunoreactivity was assessed using the lysates from transfected cells.

The polyclonal antibodies MC-20 and C1355, across both lots, recognized mouse and rat ESR1 proteins but did not react with human ESR1 protein. The monoclonal antibodies E115 and H4624 detected ESR1 proteins from all three species. E115 exhibited comparable reactivity toward human, mouse, and rat ESR1 proteins, whereas H4624 showed reduced reactivity to rat ESR1 protein (approximately 30% of the signal observed for human and mouse proteins). SP1 and F-10 antibodies selectively recognized human ESR1 protein and showed no cross-reactivity with mouse or rat proteins. No immunoreactive signals were detected in lysates from cells transfected with empty vectors or mock-transfected controls for any of the antibodies tested. Successful expression of each construct and equal protein loading were confirmed by immunoblotting with an anti-FLAG antibody (Clone 2H8).

**Fig. 2:**
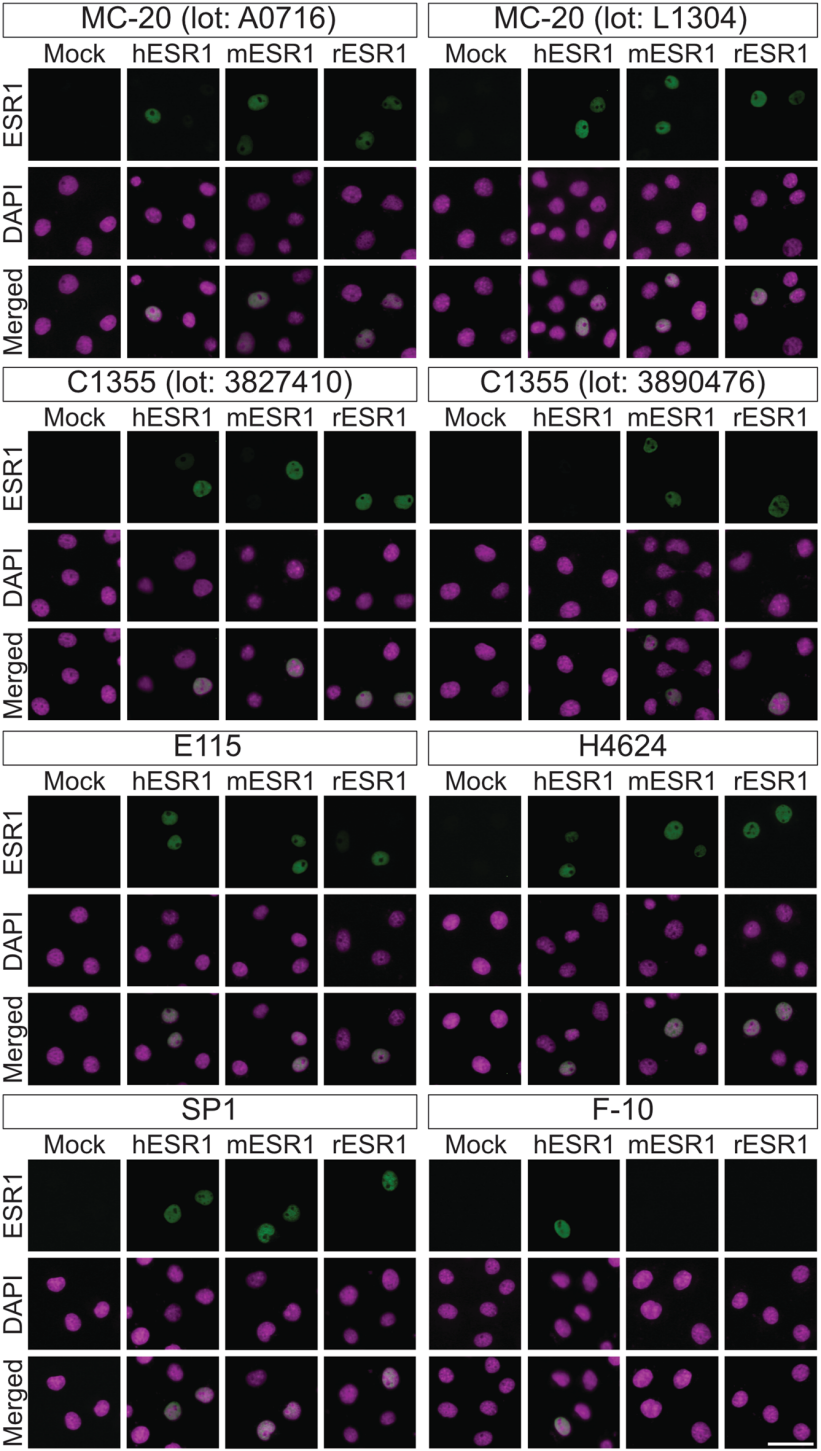
Verification of specific immunoreactivity of anti-ESR1 antibodies against human, mouse, and rat ESR1 proteins by immunocytofluorescence. Immunocytofluorescence detection of human, mouse, and rat ESR1 proteins in transfected COS-7 cells using anti-ESR1 antibodies (Clones MC-20, C1355, E115, H4624, SP1, and F-10). Two different lots were tested for each polyclonal antibody: MC-20 (Lot Nos. A0716 and L1304) and C1355 (Lot Nos. 3827410 and 3890476). “h,” “m,” and “r” indicate human, mouse, and rat, respectively. Mock-transfected cells (mock) served as negative controls. Images of human ESR1 in the MC-20 and C1355 panel sets, and rodent ESR1 in the SP1 and F-10 panel sets were acquired with longer exposure times than other ESR1 images in their respective sets. Alexa Fluor 488 and DAPI images were pseudocolored in green and magenta, respectively. Scale bar: 50 μm. Similar results were obtained in three separate experiments (*n* = 3)

The specificity and cross-reactivity of anti-ESR1 antibodies (Clones MC-20, C1355, E115, H4624, SP1, and F-10) against human, mouse, and rat ESR1 proteins were evaluated by immunocytofluorecence using transfected COS-7 cells (Fig. 2). Expression constructs encoding full-length ESR1 proteins were introduced into the cells, and immunofluorescence images were acquired with adjusted exposure times. Nuclear immunoreactive signals were detected with the polyclonal antibodies MC-20 and C1355 in cells expressing mouse and rat ESR1 constructs, irrespective of product lots. Notably, MC-20 antibodies from different lots were also able to detect human ESR1 protein when the exposure time was extended. In contrast, for the C1355 antibody, Lot No. 3827410 yielded detectable signals for human ESR1 protein under prolonged exposure but Lot No. 3890476 did not. The monoclonal antibodies E115, H4624, and SP1 produced nuclear signals in cells expressing human, mouse, and rat ESR1 proteins. However, SP1 signals were weaker in cells expressing mouse and rat ESR1 proteins compared with those expressing human protein, requiring longer exposure times for detection. The F-10 antibody produced nuclear signals exclusively in cells expressing human ESR1 protein. No immunofluorescence signals were observed in mock-transfected cells for any of the antibodies tested.

### Immunohistochemical staining of rodent uterine sections

Given that the antibody clones MC-20, C1355, E115, and H4624 exhibited strong and specific immunoreactivity against rodent ESR1 proteins, we evaluated their suitability for immunohistochemical applications. Uterine sections were initially employed to develop and optimize the staining protocol, as *Esr1* mRNA is abundantly expressed in the uteri of both mice and rats (Hattori et al. 2015; Ishii and Sakuma 2011).

According to previous immunohistochemical and in situ hybridization studies (Choijookhuu et al. 2022; Furuminato et al. 2023; Mowa and Iwanaga 2000; Pelletier et al. 2000; Sar and Parikh 1989; Shughrue et al. 1998; Yamamoto and Korach 1989), ESR1/*Esr1* is expressed in all epithelial cells and most stromal and smooth muscle cells of rodent uteri. Expression levels are highest in epithelial cells, moderate to high in stromal cells, and comparatively lower in smooth muscle cells. Accordingly, the following criteria were established to evaluate specific immunostaining of ESR1 proteins in uterine sections: epithelial cells should exhibit strong staining, stromal cells moderate to strong staining, and smooth muscle cells weak to moderate staining.

**Fig. 3:**
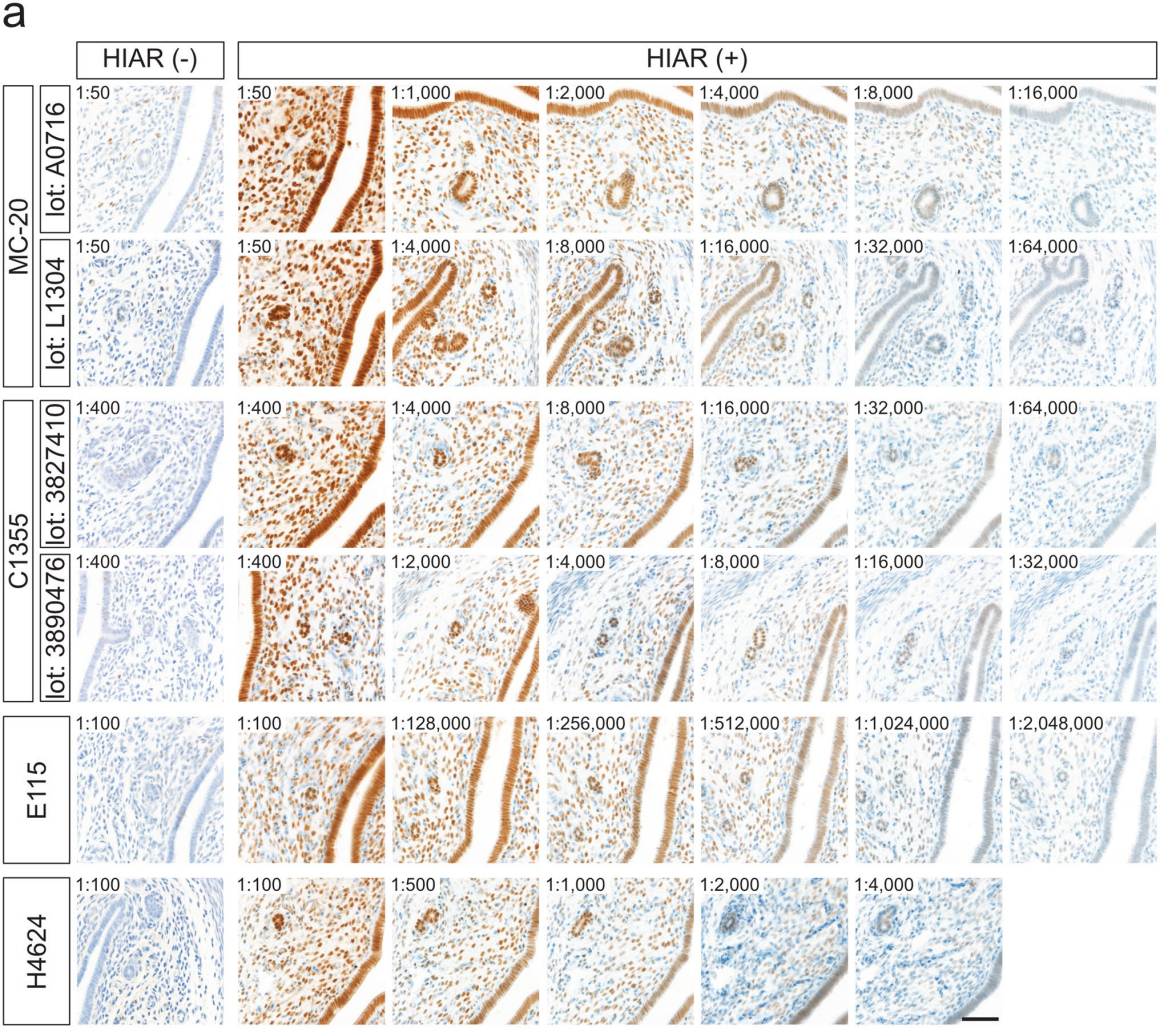

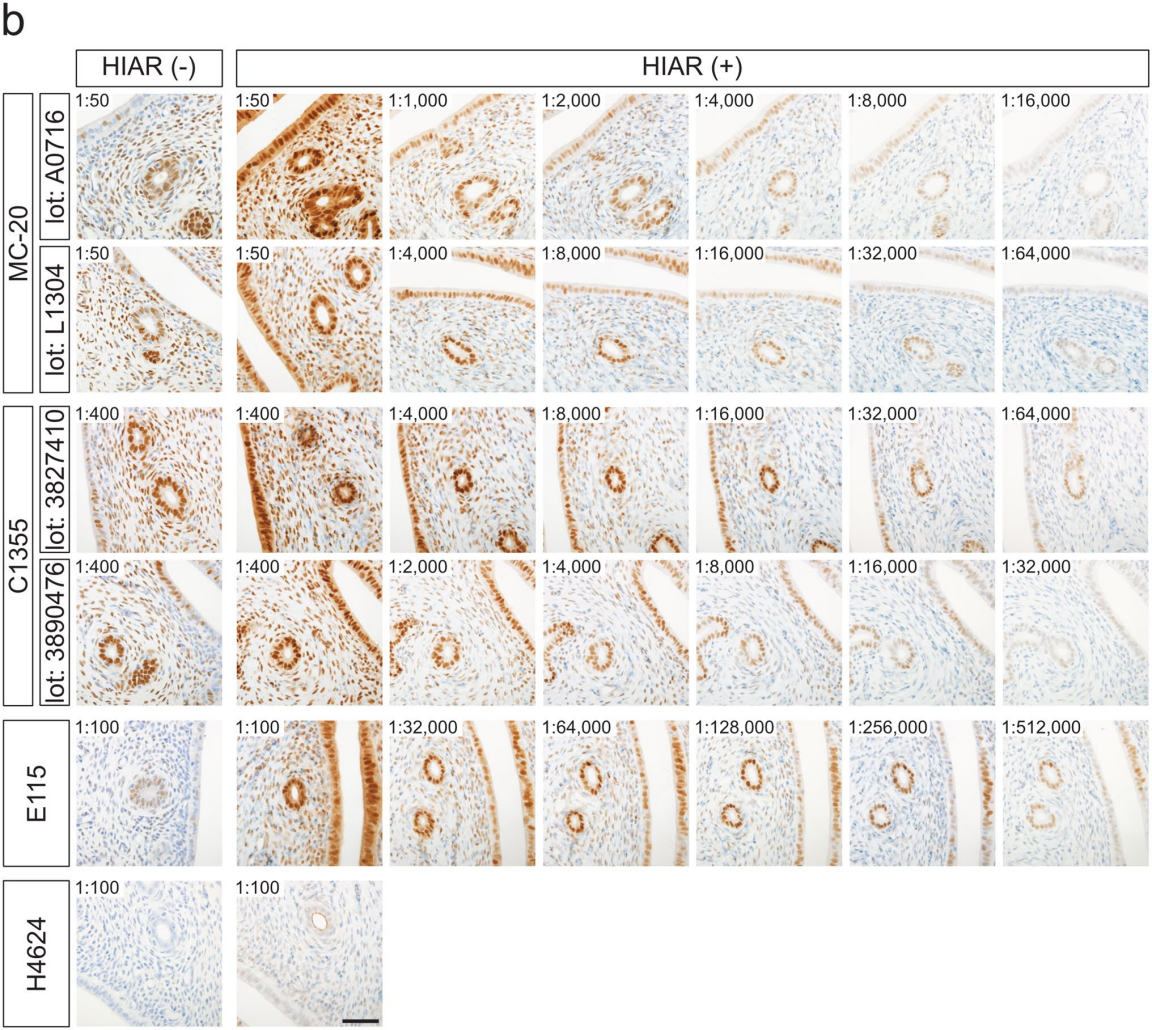
Effects of HIAR and anti-ESR1 antibody dilutions on the immunohistochemical detection of ESR1 in paraffin-embedded uterine sections from mice and rats. Paraffin-embedded uterine sections from mice (**a**) and rats (**b**) were immunostained using anti-ESR1 antibodies (Clones MC-20, C1355, E115, and H4624). Two different lots were tested for each polyclonal antibody: MC-20 (Lot Nos. A0716 and L1304) and C1355 (Lot Nos. 3827410 and 3890476). HIAR was performed by autoclaving sections at 121 °C for 10 min in a citrate-based buffer (pH 6.0). The leftmost panels show sections without HIAR [HIAR(−)], while the remaining panels show sections subjected to HIAR [HIAR(+)]. Each anti-ESR1 antibody stock solution was serially diluted and applied to the sections. The dilution ratio is indicated in the upper left corner of each panel. Scale bars: 50 μm. Similar staining patterns were observed in three independent experiments (*n* = 3)

The effects of HIAR on immunohistochemical staining were assessed using paraffin-embedded uterine sections from mice and rats (Fig. 3). Sections were autoclaved in a citrate-based buffer (pH 6.0) at 121 °C for 10 min. In paraffin-embedded mouse uterine sections, HIAR was indispensable for successful immunohistochemical detection with all antibodies tested (Fig. 3a). In rat uterine sections, immunostaining with the MC-20 and C1355 antibodies yielded detectable signals both with and without HIAR; however, signal intensity was markedly enhanced following HIAR treatment (Fig. 3b). In contrast, the E115 antibody produced only faint to weak staining in the absence of HIAR, whereas HIAR significantly improved both signal intensity and clarity. Although HIAR was essential for the H4624 antibody to generate adequate immunoreactive signals in mouse uterine sections, it failed to produce sufficient staining in rat uterine sections regardless of HIAR treatment.

Optimal antibody dilutions for the immunohistochemical detection of ESR1 proteins were determined by serially diluting stock antibody solutions. Specific immunoreactivity in uterine tissues was assessed on the basis of the staining criteria described above.

Paraffin-embedded mouse uterine sections were incubated with the following serial dilutions: 1:50 to 1:64,000 for MC-20 (Lot No. A0716), 1:50 to 1:128,000 for MC-20 (Lot No. L1304), 1:400 to 1:256,000 for C1355 (Lot No. 3827410), 1:400 to 256,000 for C1355 (Lot No. 3890476), 1:100 to 1:4,096,000 for E115, and 1:100 to 1:16,000 for H4624. Representative images from selected dilution steps are shown in Fig. 3a. The optimal dilutions for immunohistochemical detection in mouse uterine sections were determined to be 1:4000 for MC-20 (Lot No. A0716), 1:16,000 for MC-20 (Lot No. L1304), 1:16,000 for C1355 (Lot No. 3827410), 1:8000 for C1355 (Lot No. 3890476), 1:512,000 for E115, and 1:1000 for H4624.

Similarly, paraffin-embedded rat uterine sections were treated with the following dilution ranges: 1:50 to 1:32,000 for MC-20 (Lot No. A0716), 1:50 to 1:128,000 for MC-20 (Lot No. L1304), 1:400 to 1:128,000 for C1355 (Lot No. 3827410), 1:400 to 1:128,000 for C1355 (Lot No. 3890476), and 1:100 to 1:2,048,000 for E115. As shown in Fig. 3b, the optimal dilutions for rat uterine sections were 1:4000 for MC-20 (Lot No. A0716), 1:16,000 for MC-20 (Lot No. L1304), 1:16,000 for C1355 (Lot No. 3827410), 1:8000 for C1355 (Lot No. 3890476), and 1:128,000 for E115. H4624 failed to meet the staining criteria because of insufficient signal intensity.

**Fig. 4:**
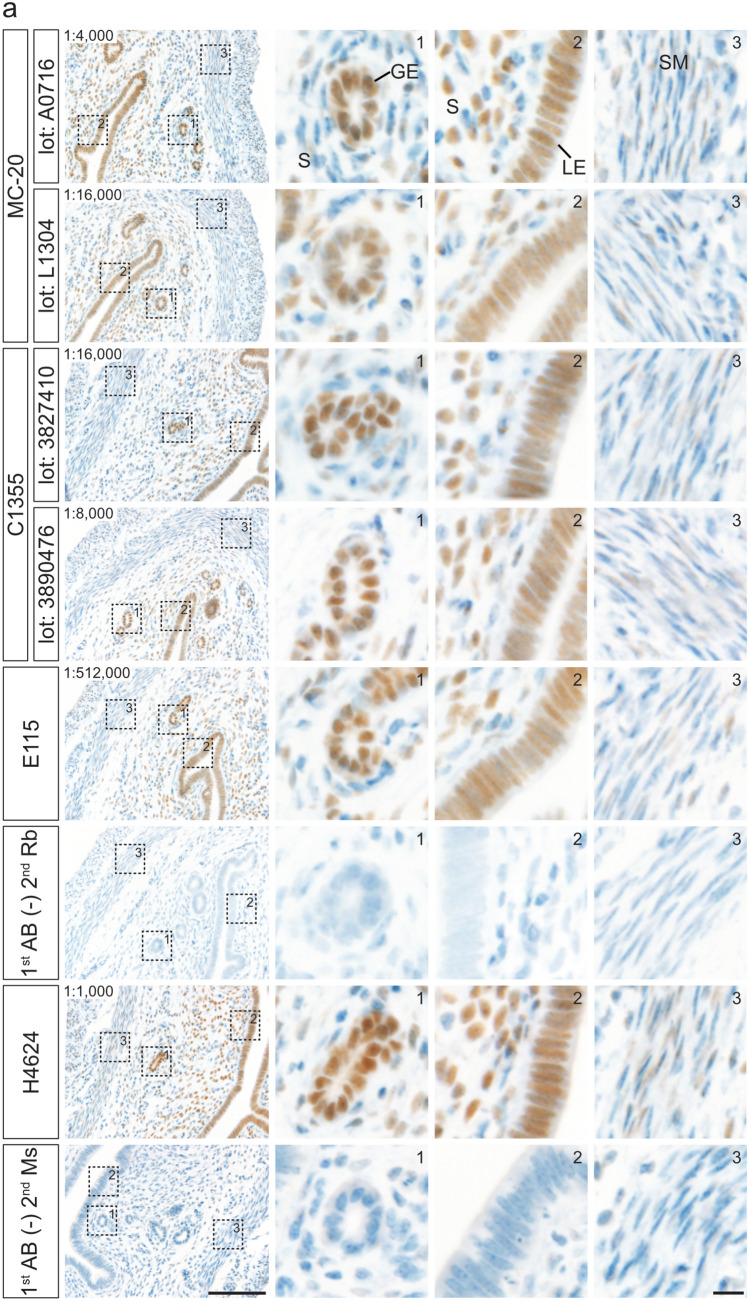

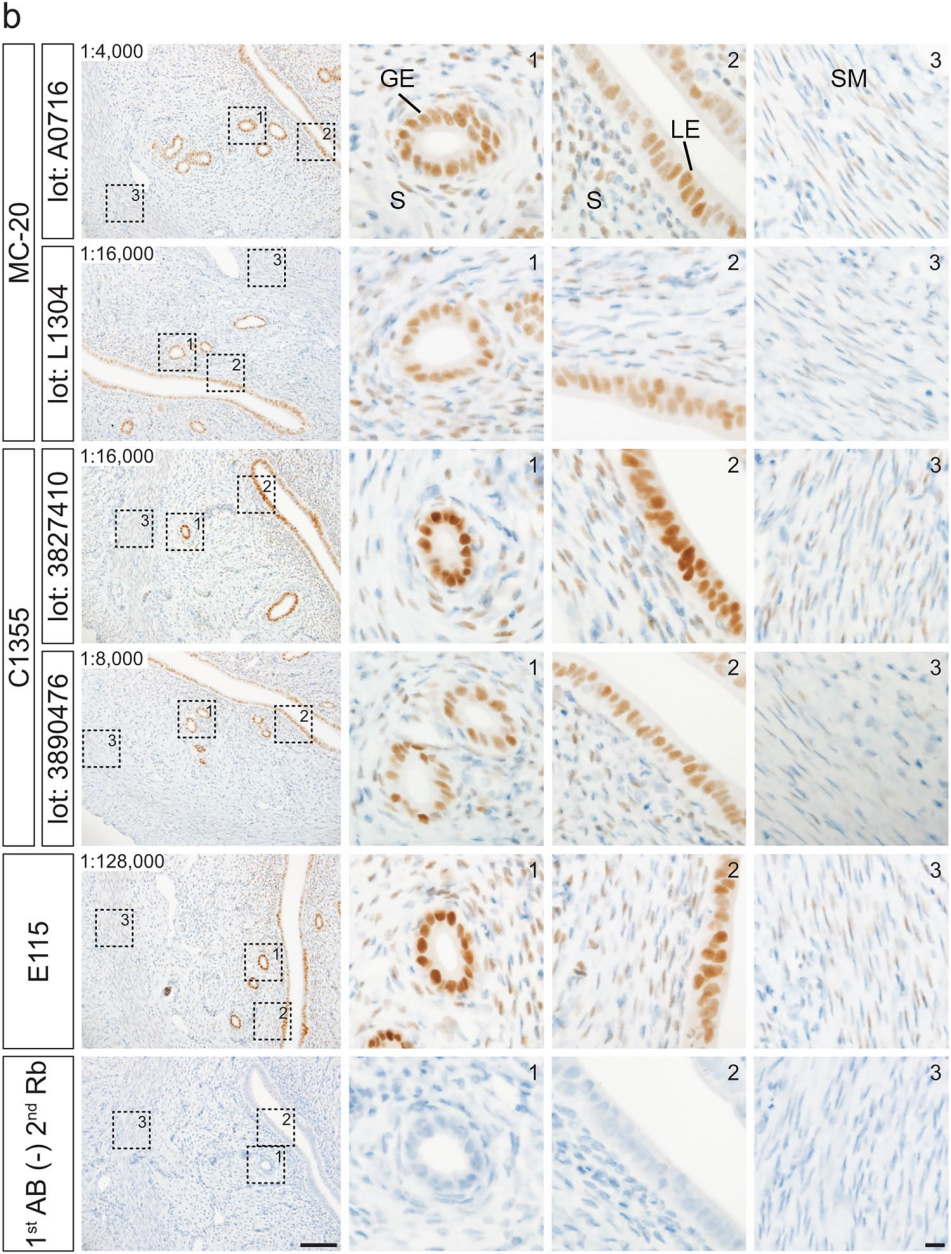
Immunohistochemical staining of mouse and rat ESR1 proteins in paraffin-embedded uterine sections under optimized conditions. Paraffin-embedded uterine sections from mice (**a**) and rats (**b**) were immunostained using anti-ESR1 antibodies (Clones MC-20, C1355, E115, and H4624) under optimized conditions. Two different lots were tested for each polyclonal antibody: MC-20 (Lot Nos. A0716 and L1304) and C1355 (Lot Nos. 3827410 and 3890476). The three right panels show higher magnification views of the boxed regions in the leftmost panels. Panels 1–3 contain glandular epithelial cells (GE) and stromal cells (S), luminal epithelial cells (LE) and stromal cells (S), and smooth muscle cells (SM), respectively. The dilution ratio is indicated in the upper left of each leftmost panel. 1st AB(−), omission of primary antibody reaction. Scale bars: 100 μm in left panels and 10 μm in right panels. Similar staining patterns were obtained in three independent experiments (*n* = 3)

Figure 4 presents representative immunostaining results in paraffin-embedded uterine sections under the optimized conditions, with Fig. 4a showing mouse tissues and Fig. 4b showing rat tissues. Glandular and luminal epithelial cells exhibited strong staining, most stromal cells showed moderate to strong staining, and most smooth muscle cells were weakly stained. The immunostaining profiles obtained with the four antibodies were largely comparable.

### Immunohistochemical staining of rodent ovarian sections

Ovarian sections were subsequently used to develop and optimize both immunohistochemistry and dual immunohistofluorescence protocols, as mouse and rat ovaries are known to express high levels of *Esr1* and *Esr2* mRNAs (Hattori et al. 2015, 2021; Ishii and Sakuma 2011; Ozawa et al. 2022). Previous immunohistochemical and in situ hybridization studies revealed that ESR1/*Esr1* is moderately expressed in many, though not all, theca and stromal cells of rodent ovaries (Hishikawa et al. 2003; Mowa and Iwanaga 2000; Pelletier et al. 2000; Sar and Welsch 1999). Accordingly, the staining criteria for ESR1 immunohistochemistry in ovarian sections were defined as moderate staining signals in most theca and stromal cells.

**Fig. 5:**
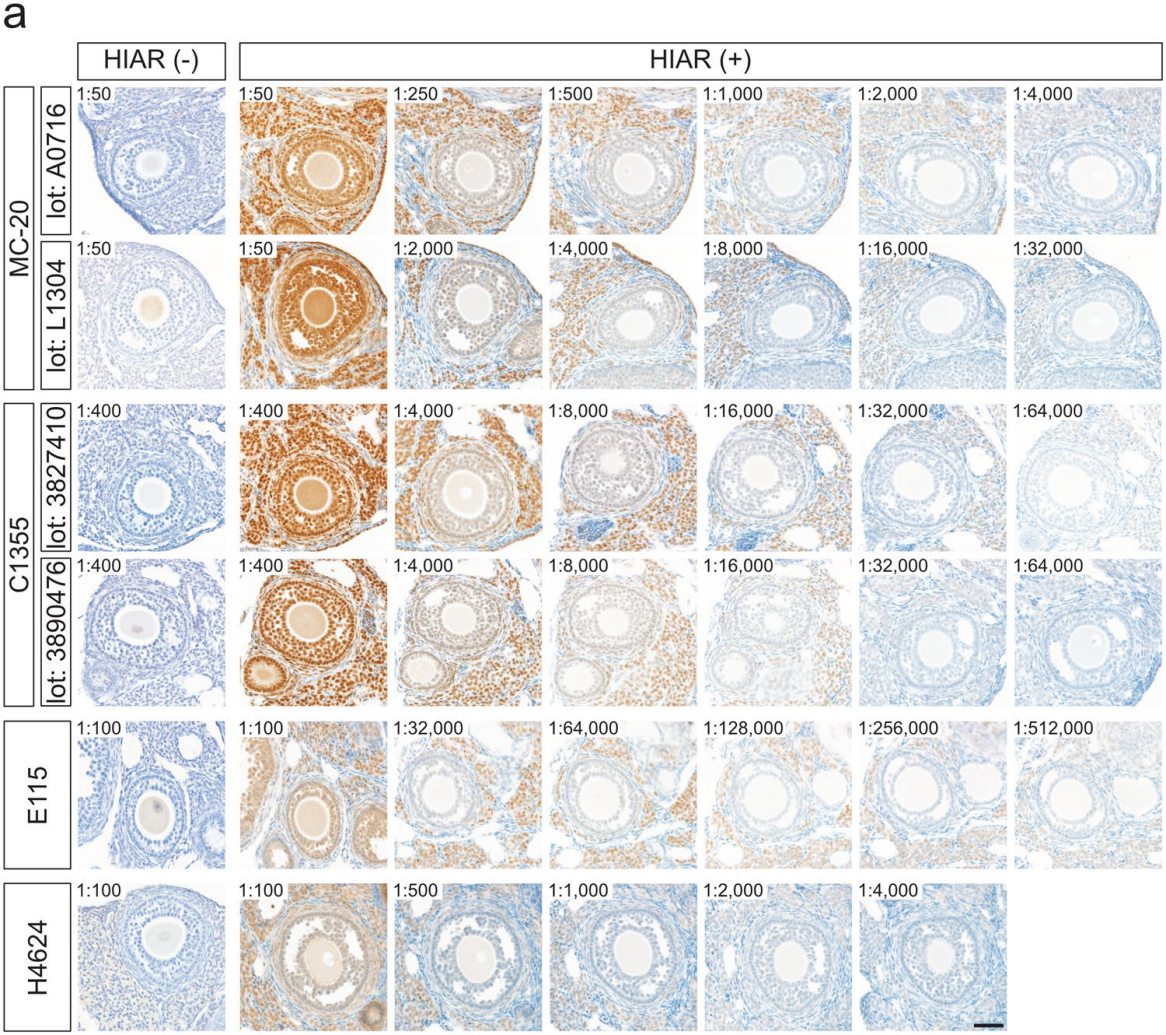

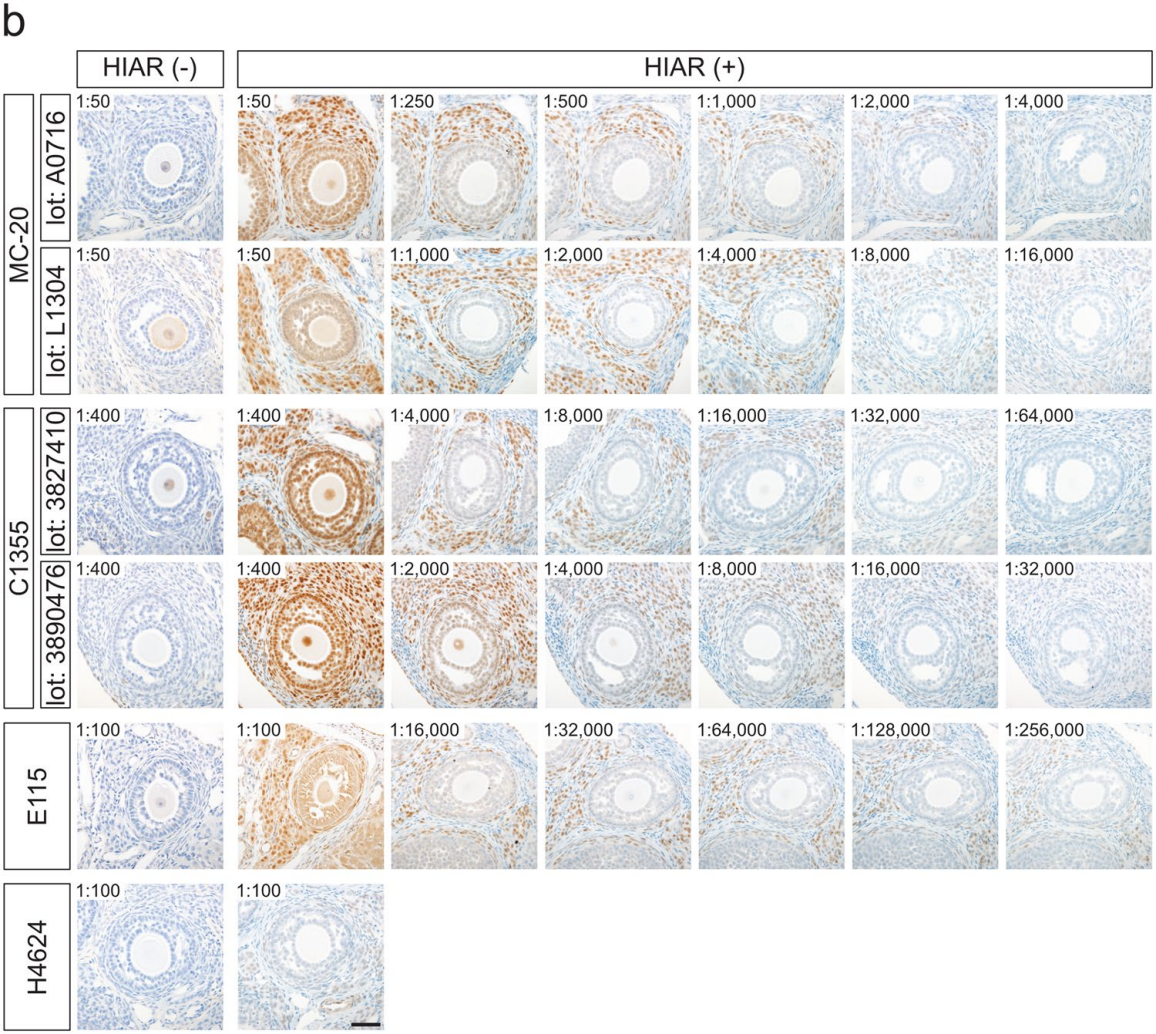
Effects of HIAR and anti-ESR1 antibody dilutions on immunohistochemical staining of mouse and rat ESR1 proteins in paraffin-embedded ovarian sections. Paraffin-embedded ovarian sections from mice (**a**) and rats (**b**) were immunostained using anti-ESR1 antibodies (Clones MC-20, C1355, E115, and H4624). Two different lots were tested for each polyclonal antibody: MC-20 (Lot Nos. A0716 and L1304) and C1355 (Lot Nos. 3827410 and 3890476). HIAR was performed by autoclaving sections at 121 °C for 10 min in a citrate-based buffer (pH 6.0). The leftmost panels show sections without HIAR [HIAR(−)], while the remaining panels show sections subjected to HIAR [HIAR(+)]. Each anti-ESR1 antibody stock solution was serially diluted and applied to the sections. The dilution ratio is indicated in the upper left of each panel. Scale bars: 50 μm. Similar staining patterns were observed in three independent experiments (*n* = 3)

The effects of HIAR and antibody dilution on staining performance were evaluated in paraffin-embedded ovarian sections from mice and rats (Fig. 5). For all antibodies tested, HIAR was essential to achieve adequate immunoreactivity in both mouse and rat ovarian sections.

Serial dilutions of each antibody were applied to paraffin-embedded ovarian sections to determine optimal working concentrations. In mouse ovarian sections (Fig. 5a), the optimal dilutions were as follows: 1:1000 to 1:2000 for MC-20 (Lot No. A0716), 1:8000 for MC-20 (Lot No. L1304), 1:16,000 for C1355 (Lot Nos. 3827410 and 3890476), 1:128,000 to 1:256,000 for E115, and 1:1000 for H4624. In rat ovarian sections (Fig. 5b), the optimal dilutions were 1:1000 for MC-20 (Lot No. A0716), 1:4000 for MC-20 (Lot No. L1304), 1:16,000 for C1355 (Lot No. 3827410), 1:8000 for C1355 (Lot No. 3890476), and 1:64,000 to 1:128,000 for E115. The H4624 antibody failed to produce sufficient immunoreactive signals in rat ovarian sections, even after HIAR treatment.

**Fig. 6:**
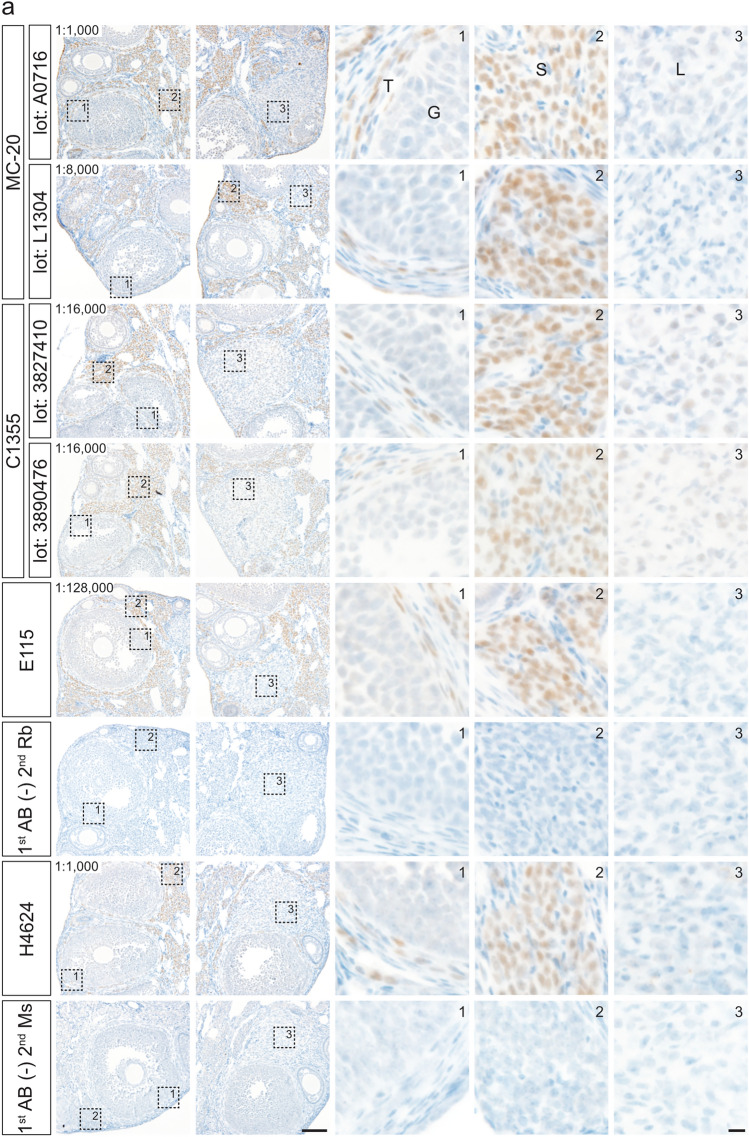

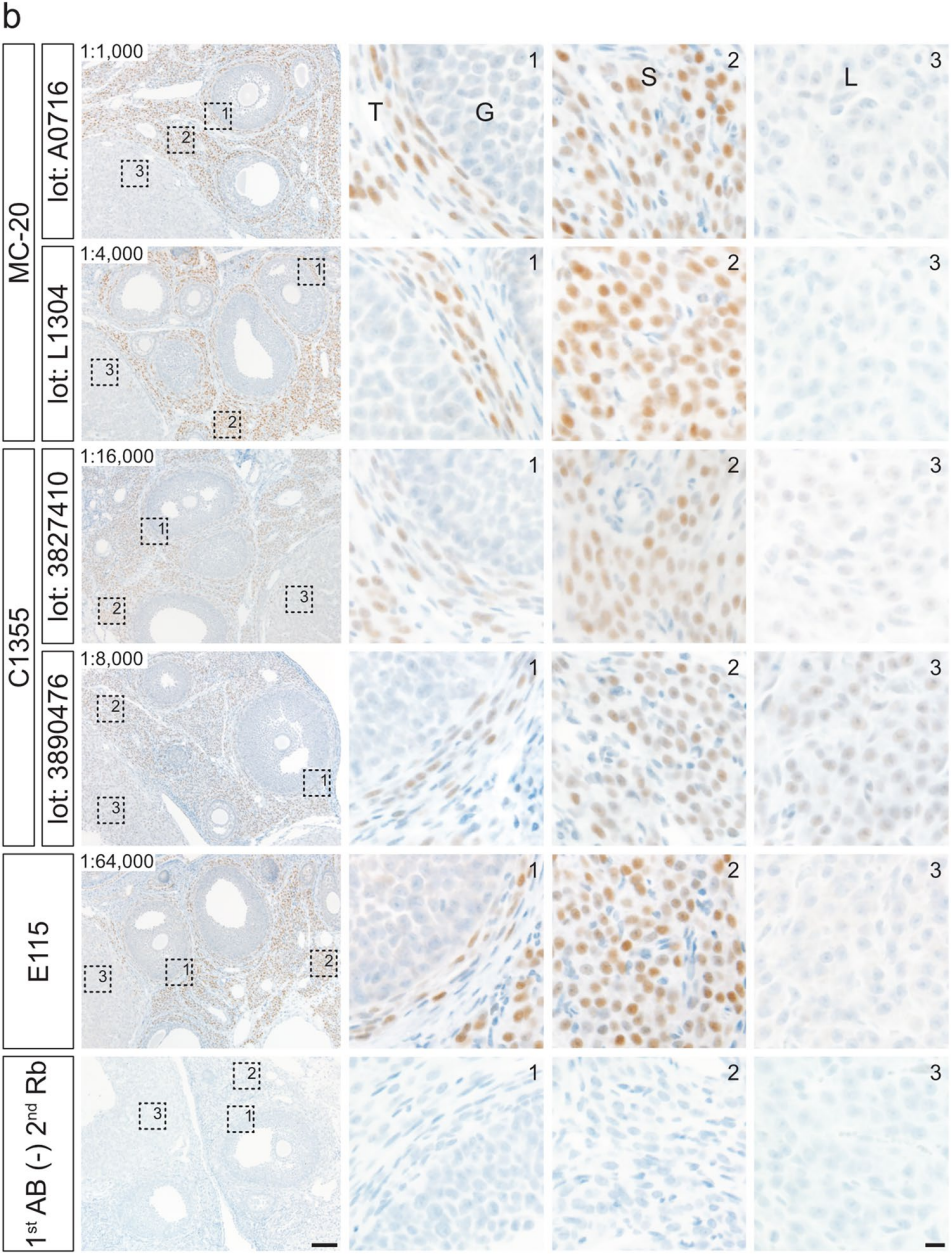
Immunohistochemical staining of mouse and rat ESR1 proteins in paraffin-embedded ovarian sections under optimized conditions. Paraffin-embedded ovarian sections from mice (**a**) and rats (**b**) were immunostained using anti-ESR1 antibodies (Clones MC-20, C1355, E115, and H4624) under optimized conditions. Two different lots were tested for each polyclonal antibody: MC-20 (Lot Nos. A0716 and L1304) and C1355 (Lot Nos. 3827410 and 3890476). The three right panels show higher magnification views of the boxed regions in the two left (**a**) and leftmost (**b**) panels. Panels 1–3 contain granulosa cells (G) and theca cells (T), stromal cells (S), and luteal cells (L), respectively. The dilution ratio is indicated in the upper left of each leftmost panel. 1st AB(−), omission of primary antibody reaction. Scale bars: 100 μm in left panels and 10 μm in right panels. Similar staining patterns were obtained in three independent experiments (*n* = 3)

Under optimized conditions (Fig. 6), immunohistochemistry using antibody clones MC-20, C1355, E115, and H4624 revealed moderate immunoreactivity in theca and stromal cells. ESR1 expression was notably heterogeneous within these cell populations. No immunostaining was detected in granulosa cells or oocytes. Luteal cells exhibited weak staining exclusively with the C1355 antibody, while the other antibodies yielded no detectable signal in these cells.

### Dual immunohistofluorescence of rodent ovarian sections for ESR1 and ESR2 proteins

**Fig. 7:**
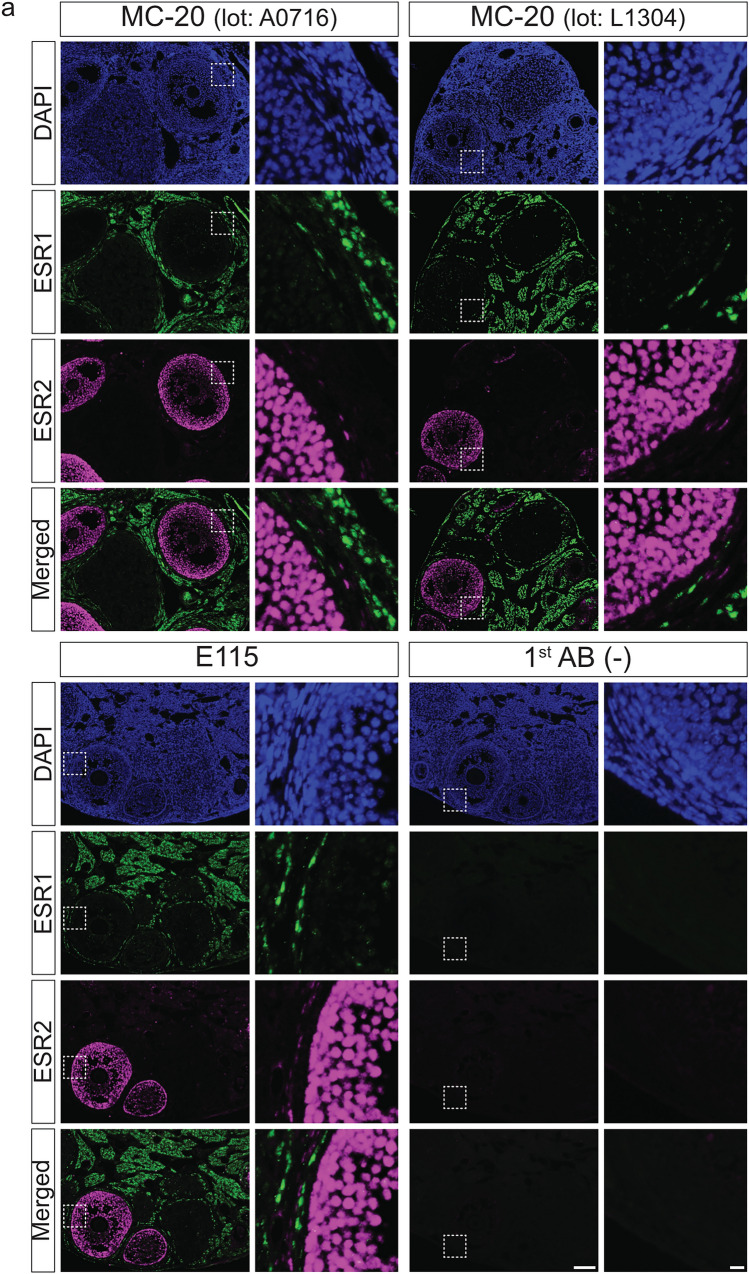

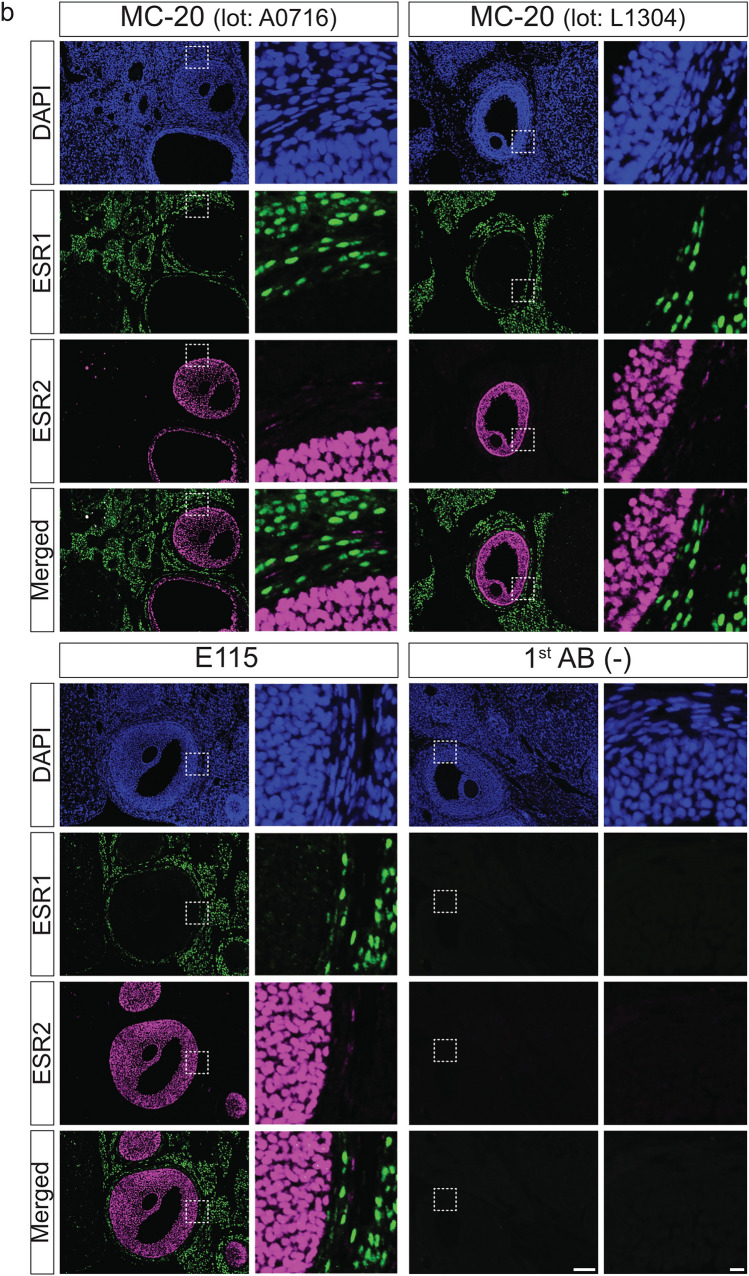
Dual immunohistofluorescence detection of ESR1 and ESR2 proteins in paraffin-embedded ovarian sections from mice and rats. Paraffin-embedded ovarian sections from mice (**a**) and rats (**b**) were dual-immunostained using an anti-ESR2 antibody (Clone PPZ0506) in combination with rabbit-derived anti-ESR1 antibodies (Clones MC-20 and E115). The right panels show higher magnification views of the boxed regions in the left panels. Alexa Fluor 488, Alexa Fluor 568, and DAPI images were pseudocolored in green, magenta, and blue, respectively. 1st AB(−), omission of primary antibody reactions. Scale bars: 100 μm in left panels and 10 μm in right panels. Similar results were obtained in three independent experiments (*n* = 3)

To investigate the co-localization of ESR1 and ESR2 proteins in paraffin-embedded ovarian sections, dual immunohistofluorescence was performed using a mouse monoclonal anti-ESR2 antibody (Clone PPZ0506) in combination with rabbit-derived anti-ESR1 antibodies (Clones MC-20 and E115) (Fig. 7). These antibodies were selected on the basis of their specificity, cross-reactivity, and host species compatibility. Because HIAR can exacerbate tissue autofluorescence, sections were exposed to LED illumination using a TiYO autofluorescence quenching illuminator (Tsuneoka et al. 2022) in a bleaching solution (Du et al. 2019) after antigen retrieval, to minimize background fluorescence.

Preliminary immunohistofluorescence experiments were conducted with reference to the optimal antibody dilutions determined by the immunohistochemical analysis described above. MC-20 (Lot No. A0716) was tested at dilutions ranging from 1:500 to 1:4000, with 1:1000 used for both mice and rats. MC-20 (Lot No. L1304) was evaluated at dilutions from 1:2000 to 1:8000, and applied at 1:4000 for mice and 1:2000 for rats. E115 was tested at dilutions ranging from 1:16,000 to 1:128,000, with 1:64,000 used for both species. The PPZ0506 antibody was used at a dilution of 1:4000 (Hattori et al. 2021; Ozawa et al. 2022).

Dual immunohistofluorescence assays for ESR1 and ESR2 yielded clear and distinct signals in ovarian sections from both mice (Fig. 7a) and rats (Fig. 7b). Strong nuclear ESR2 immunoreactivity was observed in granulosa cells. Additionally, moderate and dispersed staining was noted in theca externa layers. ESR2 signals in theca interna layers were sparse and faint, rendering detection difficult. MC-20 and E115 anti-ESR1 antibodies yielded nuclear immunoreactivity in both theca and stromal cells. Consistent with the immunohistochemical findings described above, ESR1 expression was heterogeneous across these cell populations. Clear co-localization of ESR1 and ESR2 within the same cells was rarely observed.

## Discussion

In the present study, we evaluated the specificity and cross-reactivity of six commercially available anti-ESR1 antibodies (Clones MC-20, C1355, E115, H4624, SP1, and F-10) against mouse and rat ESR1 proteins. On the basis of these findings, four antibodies (MC-20, C1355, E115, and H4624) were selected for further immunohistochemical validation, as they demonstrated specific and cross-reactive binding to rodent ESR1 proteins. Using these antibodies, we attempted to establish and optimize immunohistochemical staining protocols for paraffin-embedded uterine and ovarian sections. Through this process, MC-20 and E115 were found to be suitable for detecting ESR1 in both uterine and ovarian tissues, C1355 was effective for uterine tissue staining, and H4624 was applicable only in mouse tissues. Furthermore, we established a dual immunohistofluorescence method for ESR1 and ESR2 proteins in paraffin-embedded ovarian sections from mice and rats, employing the mouse monoclonal anti-ESR2 antibody PPZ0506 in combination with the selected rabbit-derived anti-ESR1 antibodies (Clones MC-20 and E115).

The specificity and cross-reactivity of anti-ESR1 antibodies were clearly demonstrated by immunoblotting, which revealed distinct and specific reactivity toward human, mouse, and rat ESR1 proteins. In contrast, immunocytofluorescence analysis showed a generally similar pattern of reactivity; however, some discrepancies were observed relative to the immunoblotting results. These findings indicate that antibody specificity and cross-reactivity should be assessed using multiple complementary approaches. Moreover, the MC-20 and C1355 polyclonal antibodies exhibited lot-to-lot variability in both reactivity and titer. Reactivity of the C1355 antibody to human ESR1 protein varied depending on the antibody lot in immunocytofluorescence, and differences in antibody titer were observed between different lots in immunohistochemistry. These results highlight the critical importance of validating each antibody lot when using polyclonal antibodies, as such variability can substantially affect the reliability and reproducibility of experimental outcomes.

We previously optimized immunohistochemical staining protocols for ESR2 proteins using the PPZ0506 monoclonal antibody (Hattori et al. 2021; Morishita et al. 2023a, b; Ozawa et al. 2022). Given that successful ESR2 immunohistochemistry required HIAR and careful optimization of the antibody dilution, we investigated whether similar conditions would enhance the detection of ESR1 proteins in rodent tissues. Our present results demonstrate that both HIAR and an appropriate antibody dilution were essential for reliable immunohistochemical detection of ESR1 in paraffin-embedded sections from mice and rats. Without HIAR, the H4624 antibody failed to exhibit specific immunoreactivity; moreover, immunostaining with MC-20, C1355, and E115 antibodies was inconsistent, varying by tissue type and species. In contrast, when HIAR was applied, all four antibodies produced clear staining across tissues and species, even at low antibody concentrations. During our prior optimization efforts (Morishita et al. 2023a, b; Ozawa et al. 2022), we also identified two key factors that improved staining quality: the use of a commercial Mouse-on-Mouse Blocking Buffer (Abcam) to suppress endogenous IgG signals in mouse-on-mouse immunohistochemistry, and the application of an HRP-conjugated polymer secondary antibody to improve the signal-to-noise ratio. These strategies were therefore incorporated into the current ESR1 staining protocol.

Previous studies have reported that microwave-mediated HIAR improves ESR1 staining in frozen sections of monkey oviductal fimbriae (Koji 2025; Slayden et al. 1995). Without HIAR, ESR1 immunoreactivity was clearly observed in stromal cells but was barely detectable in the epithelium, whereas HIAR markedly enhanced epithelial ESR1 staining. In the present study, we employed autoclave-mediated HIAR instead of microwave treatment, as microwave-mediated HIAR is insufficient for ESR2 immunohistochemistry (Hattori et al. 2021), and this study focuses on the co-staining of ESR1 and ESR2. Although our section processing, HIAR method, and antibody selection differ from those used in the previous studies, we observed similar improvements in ESR1 staining following HIAR in rat uterine sections. Furthermore, we demonstrated that HIAR is essential for ESR1 immunostaining in the other sections.

Four antibodies (Clones MC-20, C1355, E115, and H4624) were selected on the basis of their specificity and cross-reactivity with mouse and rat ESR1 proteins. Among them, the H4624 antibody showed immunohistochemical utility exclusively in mouse tissues, consistent with its low cross-reactivity to rat ESR1 protein observed in immunoblotting analyses. Immunohistochemical analyses using all four antibodies revealed largely comparable staining profiles in uterine sections. In ovarian sections, although the overall staining patterns were similar across antibodies, only the C1355 antibody yielded weak staining in luteal cells. Given that both MC-20 and C1355 antibodies were raised against the C-terminal regions of ESR1, the possibility that this discrepancy arises from ESR1 splice variants (Ishii et al. 2023) can be excluded. Instead, the weak luteal cell staining observed with C1355 is presumed to result from non-specific reactivity. Therefore, the E115 antibody represents a suitable alternative to MC-20 for immunohistochemical detection of ESR1 proteins in mouse and rat tissues.

The lack of reliable anti-ESR2 antibodies has long posed a challenge in accurately determining the distribution profiles of ESR2 proteins. The recent discovery of a highly specific anti-ESR2 antibody (Clone PPZ0506), combined with optimized immunohistochemical protocols, has markedly enhanced the ability to localize ESR2 expression in tissues (Andersson et al 2017; Hattori et al. 2021; Hawse et al. 2020; Ishii et al. 2019; Morishita et al. 2023a, b; Ozawa et al. 2022; Schröder et al. 2022). Contrary to earlier assumptions that ESR2 is broadly expressed, emerging evidence indicates that ESR2 exhibits a more spatially restricted distribution. Furthermore, interspecies variation in ESR2 expression profiles has been documented, suggesting evolutionary divergence in estrogen receptor signaling pathways.

In vitro studies have demonstrated that ESR2 can interact with ESR1 and modulate its transcriptional activity (Cowley et al. 1997; Pettersson et al. 1997), highlighting the importance of investigating their co-expression in situ to better understand the mechanisms underlying estrogen receptor crosstalk. To this end, we employed dual immunohistofluorescence in rodent ovaries with high expression levels of both *Esr1* and *Esr2*. Both MC-20 and E115 anti-ESR1 antibodies were applicable to double immunohistofluorescence with the specific anti-ESR2 antibody PPZ0506. Using this approach, we obtained clear and characteristic immunofluorescent signals in ovarian sections from both mice and rats. Notably, co-localization of ESR1 and ESR2 within the same cells was rare, suggesting distinct functional roles and spatial segregation of these receptors in rodent ovarian tissues.

ESR2 protein is expressed in the brain and ovary of mice, and in the brain, ovary, and ventral and dorsal lobes of the prostate of rats (Hattori et al. 2021; Morishita et al. 2023a, b; Ozawa et al. 2022). Among these tissues, the brain is the only site, aside from the ovary, where ESR1 and ESR2 proteins may co-localize. Therefore, future studies should apply dual immunohistofluorescence for ESR1 and ESR2 proteins in rodent brain tissues to investigate their potential co-localization in vivo. Furthermore, to elucidate possible interactions between ESR1 and ESR2 proteins, in vivo analyses using techniques such as fluorescence resonance energy transfer and proximity ligation assays (Fredriksson et al. 2002; Kenworthy 2001) with labeled anti-ESR1 and anti-ESR2 antibodies will be necessary.

## Conclusions

We optimized immunohistochemical protocols for the specific detection of rodent ESR1 protein by employing rigorously validated anti-ESR1 antibodies and established dual immunohistofluorescence methods for the simultaneous visualization of ESR1 and ESR2 proteins with specific anti-ESR1 and anti-ESR2 antibodies. Little co-localization of ESR1 and ESR2 proteins was observed in the ovaries of both mice and rats. Given that both ESR1 and ESR2 proteins are also expressed in the rodent brain, applying these dual immunohistofluorescence techniques to brain tissues will be essential for determining whether ESR1 and ESR2 proteins co-localize and potentially interact within the central nervous system. We anticipate that the protocols described here will serve as robust tools to reliably analyze the distribution of rodent ESR1 protein and potential co-localization of rodent ESR1 and ESR2 proteins for future studies, ultimately advancing our understanding of the intricate mechanisms of estrogen signaling mediated by ESR1 and ESR2.

## Data Availability

The data that support the findings of this study are available from the corresponding author upon reasonable request.

## Abbreviations

DAPI: 4′,6-Diamidino-2-phenylindole
ER: Estrogen receptor
ERα: Estrogen receptor α
ERβ: Estrogen receptor β
HIAR: Heat-induced antigen retrieval
HRP: Horseradish peroxidase
PBS: Phosphate-buffered saline
PBST: PBS containing Triton X-100
